# Private, public, and bottled drinking water: Shared contaminant-mixture exposures and effects challenge

**DOI:** 10.1016/j.envint.2024.109220

**Published:** 2024-12-19

**Authors:** Paul M. Bradley, Kristin M. Romanok, Kelly L. Smalling, Stephanie E. Gordon, Bradley J. Huffman, Katie Paul Friedman, Daniel L. Villeneuve, Brett R. Blackwell, Suzanne C. Fitzpatrick, Michael J. Focazio, Elizabeth Medlock-Kakaley, Shannon M. Meppelink, Ana Navas–Acien, Anne E. Nigra, Molly L. Schreiner

**Affiliations:** a U.S. Geological Survey, Columbia, SC, USA; b U.S. Geological Survey, Lawrenceville, NJ, USA; c U.S. Geological Survey, Kearneysville, WV, USA; d U.S. Environmental Protection Agency, Durham, NC, USA; e U.S. Environmental Protection Agency, Duluth, MN, USA; f U.S. Food and Drug Administration, Silver Spring, MD, USA; g U.S. Geological Survey, Reston, VA, USA; h U.S. Geological Survey, Iowa City, IA, USA; i Department of Environmental Health Sciences, Columbia University, New York, NY, USA

**Keywords:** Point of use, Drinking water, Public supply, Private supply, Bottled water, Contaminant mixtures, Organics, Inorganics

## Abstract

**Background:**

Humans are primary drivers of environmental–contaminant exposures worldwide, including in drinking-water (DW). In the United States, point-of-use DW (POU–DW) is supplied via private tapwater (TW), public-supply TW, and bottled water (BW). Differences in management, monitoring, and messaging and lack of directly–intercomparable exposure data influence the actual and perceived quality and safety of different DW supplies and directly impact consumer decision–making.

**Objectives:**

The purpose of this paper is to provide a meta-analysis (quantitative synthesis) of POU–DW contaminant–mixture exposures and corresponding potential human–health effects of private-TW, public-TW, and BW by aggregating exposure results and harmonizing apical–health–benchmark–weighted and bioactivity–weighted effects predictions across previous studies by this research group.

**Discussion:**

Simultaneous exposures to multiple inorganic and organic contaminants of known or suspected human-health concern are common across all three DW supplies, with substantial variability observed in each and no systematic difference in predicted cumulative risk between supplies. Differences in contaminant or contaminant–class exposures, with important implications for DW–quality improvements, were observed and attributed to corresponding differences in regulation and compliance monitoring.

**Conclusion:**

The results indicate that human-health risks from contaminant exposures are common to and comparable in all three DW–supplies, including BW. Importantly, this study’s target analytical coverage, which exceeds that currently feasible for water purveyors or homeowners, nevertheless is a substantial underestimation of the breadth of contaminant mixtures in the environment and potentially present in DW. Thus, the results emphasize the need for improved understanding of the adverse human-health implications of long-term exposures to low–level inorganic–/organic–contaminant mixtures across all three distribution pipelines and do not support commercial messaging of BW as a systematically safer alternative to public-TW. Regardless of the supply, increased public engagement in source-water protection and drinking–water treatment is necessary to reduce risks associated with long-term DW–contaminant exposures, especially in vulnerable populations, and to reduce environmental waste and plastics contamination.

## 1. Introduction

Humans are fundamental drivers of Earth-system change in the extant Anthropocene event (Crutzen and Stoermer, 2000; Gibbard et al., 2022). The planetary boundary or safe operating zone (Rockström et al., 2009a; Rockström et al., 2009b) for “chemical pollution” (expanded to “novel entities” to encompass anthropogenic [human-synthesized or –driven] contaminants including new substances, new forms of existing substances, and modified life forms (Steffen et al., 2015)) has remained largely undefined due to an ever–expanding, poorly-characterized, anthropogenic chemosphere (Diamond et al., 2015; Wang et al., 2020) and to the undetermined cumulative effects of characteristically complex environmental–contaminant exposures (Altenburger et al., 2015; Altenburger et al., 2018; Escher et al., 2014; Stalter et al., 2020; Tang et al., 2013) on sensitive species and life cycle periods, including human (Diamond et al., 2015; Persson et al., 2013; Rockström et al., 2009a; Steffen et al., 2015). Recent reports have highlighted planetary–boundary threats for specific contaminant concerns including plastics (Arp et al., 2021; Villarrubia-Gómez et al., 2018) and per/polyfluoroalkyl substance(s) (PFAS) (Cousins et al., 2022), while Persson et al. (2022) argue that the broader “novel entities” planetary boundary has already been breached because global production and associated environmental releases far outpace any existing global capacity for assessment and monitoring.

Despite a nascent understanding of human-health effects and concomitant underestimation of the corresponding contribution to the global–disease burden, anthropogenic–contaminant exposures have been identified as the largest environmental cause of disease and premature death in the world today (Fuller et al., 2022; Landrigan et al., 2018). Because the biological imperative for water places it at a critical nexus of anthropogenic contamination and human-health concerns, drinking water (DW; drinking/cooking water, collectively, herein) safety and sustainability are global priorities (Allaire et al., 2018; Collier et al., 2019; Debbeler et al., 2018; Doria, 2010; Doria et al., 2009; Doria, 2006; Han et al., 2023; Hu et al., 2011; Meehan et al., 2020a; Meehan et al., 2020b; Pierce and Gonzalez 2017; Rosenblum et al., 2024; Rosinger et al., 2018; Saylor et al., 2011; Villanueva et al., 2014; Zipper et al., 2020) and high–leverage points for public engagement in pollution control and mitigation actions from global to local scales (Collier et al., 2019; Debbeler et al., 2018; Faust et al., 2015; Faust et al., 2017; Han et al., 2023; Patel and Schmidt 2017; Zipper et al., 2020). However, fundamental differences in management, monitoring, and marketing profiles (Table 1); resultant imbalances in scientifically-objective versus commercial messaging; and the general lack of intercomparable, environmentally–representative exposure data influence the perceived quality and safety of different DW supplies and directly impact consumer decision–making, including public engagement in source-water protection and investment in DW–treatment improvements (Bouhlel et al., 2023; Cohen and Ray 2018; Debbeler et al., 2018; Doria et al., 2009; Hawkins 2017; Jaffee and Newman 2013; Jaffee 2020; Meehan et al., 2020a; Opel 1999; Saylor et al., 2011; U.S. Government Accountability Office 2009; Wilk 2006).

Point-of-use DW (POU-DW) is delivered to consumers in the United States (US) and globally via three general distribution “pipelines” or supply chains, comprising private tapwater (private–TW; predominantly private wells in the US), public-supply (community water supply) tapwater (public–TW), and bottled water (BW). Among these, BW supply in the US is dominated (more than 70 %) by single–serving sizes (Rodwan 2021) and, consequently, functions as a potential supplement to but not a replacement for residential or workplace TW (public or private), having limited cooking–water use and little practicality for most other domestic and workplace water uses including hygiene and sanitation. A recent report (Food and Water Watch.org 2018), citing proprietary beverage market data, indicated that public–TW, at more than 63 % of market share by volume in 2014, is the primary source for BW consumed in the US, more than double the 30 % estimated in 1994 (Allen and Darby 1994; Olson et al., 1999). The 2023 U.S. Geological Survey (USGS) BW inventory estimated that more than 64 % of BW facilities in the US were entirely public–TW sourced, with an additional 15 % sourced from a combination of public–TW and private–supply (Buchwald et al., 2023; Corson-Dosch et al., 2023).

A range of factors influence the actual and perceived quality and sustainability of all three POU–DW distribution pipelines. The breadth of anthropogenic chemicals in commercial use (Wang et al., 2020) and, thus, potentially-present or already documented in ambient (surface water, groundwater) DW–source waters (Altenburger et al., 2015; Bradley et al., 2017; de Jesus Gaffney et al., 2015; DeSimone et al., 2015b; Moschet et al., 2014; Toccalino and Hopple 2010; Toccalino et al., 2012) exceeds by orders of magnitude the number of contaminants regulated in US DW (U.S. Environmental Protection Agency 2024d, e; U.S. Food & Drug Administration 2024a, b). Growing population-driven water-use demands (Dieter et al., 2018; Maupin 2018) and diminishing pristine (un-impacted) DW resources have led inexorably to increasing reliance on intentional (direct and indirect potable) and *de facto* water reuse (Maupin et al., 2014; Nguyen et al., 2018; Rice et al., 2013; Rice et al., 2015; Rice and Westerhoff 2017; Weisman et al., 2021). Regulated and unregulated organic contaminants have been shown to persist through existing DW–treatment processes and have been documented in treated public–TW and BW prior to distribution and at the point of consumption (Bradley et al., 2023b; Conley et al., 2017; Furlong et al., 2017; Glassmeyer et al., 2017; Guelfo and Adamson 2018; Hu et al., 2016; Klarich et al., 2017; Klarich Wong et al., 2019; Olson et al., 1999; Rosenblum et al., 2024; Smalling et al., 2023; Stackelberg et al., 2004; Stackelberg et al., 2007). The quality of TW and BW can change substantially within service lines and premise plumbing and within packaging, respectively (Makris et al., 2014; Triantafyllidou and Edwards 2012a; U.S. Environmental Protection Agency 2022a, b), raising fundamental concerns about inadequate water–quality characterization at the time and place of human exposure (Bondy and Campbell 2018; Bradley et al., 2018; Bradley et al., 2020; Bradley et al., 2023b; Braun and Gray 2017). DW disinfection, which undeniably protects against high-mortality, water–borne–pathogen disease outbreaks (Reynolds et al., 2008; Richardson et al., 2007; Schoenen 2002), can degrade organoleptic (e.g., taste and odor) quality (Doria 2010; Doria et al., 2009; Doria 2006; Pierce and Gonzalez 2017) and result in genotoxic/cytotoxic disinfection byproducts (DBP) (Jeong et al., 2015; Krasner et al., 2016; Muellner et al., 2007; Pressman et al., 2010; Richardson et al., 2007; Stalter et al., 2020; Villanueva et al., 2018; Wagner and Plewa 2017; Wang et al., 2015); both are notable leverage points for BW marketing (Debbeler et al., 2018; Gorelick et al., 2011; Wilk 2006). Chemical and biological water–quality incidents are well documented and highly publicized in public-TW and, to a notably lesser extent, in BW (Akhbarizadeh et al., 2020; Brumfield and Colwell 2015; Collier et al., 2012; Craun et al., 2010; Felton 2020a, b; Hanna-Attisha et al., 2016). DW–contaminant–mixture exposures, including at low-levels, are increasingly associated with adverse human-health outcomes (Bondy and Campbell 2018; Braun and Gray 2017; Carlin et al., 2013; Collier et al., 2012; Cui et al., 2016; Gross and Birnbaum 2017; Stalter et al., 2020).

Although the above source-water and infrastructure concerns affect all three distribution pipelines to varying extents, fundamental differences in US regulation, monitoring, community–right–to–know practices, and, notably, commercial marketing between private-TW, public-TW, and BW (Table 1) also contribute to and, in the latter case, intentionally drive public perceptions of the quality and safety of different drinking-water supplies and, consequently, directly impact consumer decision–making and water-quality/-sustainability engagement (Bouhlel et al., 2023; Cohen and Ray 2018; Debbeler et al., 2018; Doria et al., 2009; Hawkins 2017; Jaffee and Newman 2013; Jaffee 2020; Meehan et al., 2020a; Opel 1999; Saylor et al., 2011; U.S. Government Accountability Office 2009; Wilk 2006). Private–TW (i.e., <25 people served, <15 service connections) is not federally regulated or systematically monitored in the US (U.S. Environmental Protection Agency 2023d). Because homeowners bear the burden for all private–supply monitoring and maintenance costs (U.S. Environmental Protection Agency 2023d), monitoring data are generally lacking (Zheng and Flanagan 2017), significant socioeconomic disparities in monitoring and maintenance exist (Flanagan et al., 2016), and broad–scope contaminant monitoring is rare (Bradley et al., 2021a). In stark contrast, public–TW is actively regulated as DW by the US Environmental Protection Agency (EPA, statutory authority), typically with state or Tribal primacy, under the Safe Drinking Water Act (SDWA) (U.S. Environmental Protection Agency 2024d, e). For public–TW, National Primary Drinking Water Regulation contaminants are routinely (as determined by USEPA’s Standardized Monitoring Framework (U.S. Environmental Protection Agency 2020)) monitored, annual consumer–confidence reports and customer accessibility are mandated (U.S. Environmental Protection Agency 2024d, e), and system–specific SDWA violation data are tracked, publicly available, and readily accessible (U.S. Environmental Protection Agency 2023a, c). Between these monitoring and regulation end–members, the Food and Drug Administration (FDA), which has no specific statutory authority to regulate DW (U.S. Government Accountability Office 2009), regulates BW as a “food” under the Food, Drugs, and Cosmetics Act (FD&C Act), with corresponding routine (weekly) sanitary (bacteriological) monitoring but infrequent (stipulated only as “at least annually”) chemical, physical, and radiological monitoring requirements for bottling facilities (U.S. Food & Drug Administration 2024a, b). BW standard of quality (SOQ, “shall not contain in excess of”) levels (U.S. Food & Drug Administration 2024a, b) are by law (21 U.S.C. § 349 1996) adapted from and, with few exceptions (e.g., lead [Pb]), equivalent to EPA National Primary Drinking Water Regulation maximum contaminant level(s) (MCL) (U.S. Environmental Protection Agency 2024d, e). Access to BW water–quality compliance monitoring data, beyond the minimal information on the package label, however, is by direct request to the BW supplier and, thus, limited and generally *post hoc* (U.S. Government Accountability Office 2009). Detailed publicly–available, quantitative assessments of BW quality historically have been restricted to scientific investigations, often without the brand identification needed for purchase decisions (e.g., Bradley et al., 2023b; Olson et al., 1999).

Importantly, BW is unique among the three distribution pipelines as a commercial product; unsurprisingly, corresponding market–driven messaging (advertising) far outweighs BW water–quality data dissemination. Commercial promotion as a safer DW alternative to private–TW or public–TW (Hawkins 2017; Olson et al., 1999) amidst heightened concerns about environmental contaminant exposures and health risks (Pape and Seo 2015; Zivin et al., 2011) has dramatically increased BW consumption in the US (Rodwan 2021) and globally (Bouhlel et al., 2023; Greene 2018). BW consumption is projected to continue increasing despite 1) a paucity of directly comparable, realistically–comprehensive BW–contaminant–exposure data (Bouhlel et al., 2023; Bradley et al., 2023b), 2) well–documented economic and environmental impacts (e.g., cost, sustainability) (Bouhlel et al., 2023; Collier et al., 2019; Knox and McDermott 2019; Olson et al., 1999; Onufrak et al., 2014; Pierce and Gonzalez 2017; Saylor et al., 2011), 3) notable contribution to the plastics planetary–boundary threat (Arp et al., 2021; Bouhlel et al., 2023; Hawkins 2011; Persson et al., 2022; Villarrubia-Gómez et al., 2018), 4) orders-of-magnitude higher micro–/nano–plastics contamination than TW (Cox et al., 2019; Kannan and Vimalkumar 2021; Mason et al., 2018; Mitrano et al., 2021; Qian et al., 2024), 5) growing concerns about micro–/nano–plastics–contaminant ingestion, biological uptake, translocation (including fetal), and toxicity (Cox et al., 2019; Kannan and Vimalkumar 2021; Medley et al., 2023), and 6) high estimated plastic-attributable US disease burden (Trasande et al., 2024).

Redressing existing water–quality–information imbalances and informing POU-DW mixture exposures and associated distal (e.g., ambient source water) and proximal (e.g., premise plumbing, point-of-use treatment, bottled-water packaging) drivers across all three DW–distribution pipelines are priorities of ongoing USGS POU-DW research collaborations with EPA, FDA, other state and federal agencies, Tribal nations and entities, universities, water utilities, communities, and residential participants (Bradley et al., 2018; Bradley et al., 2020; Bradley et al., 2021a; Bradley et al., 2021b; Bradley et al., 2022; Bradley et al., 2023a; Bradley et al., 2023b; Smalling et al., 2023). Sampling personnel; collection protocols; core target inorganic/organic/microbial analytes, methods, and laboratories; and quality–assurance/quality–control procedures are maintained to ensure intercomparability across study areas and DW–distribution pipelines. To date, private-TW and public-TW contaminant mixtures and associated drivers have been assessed by this group in a range of socioeconomic and source–water vulnerability settings across the US (Bradley et al., 2018; Bradley et al., 2020; Bradley et al., 2021a; Bradley et al., 2021b; Bradley et al., 2022; Bradley et al., 2023a; Smalling et al., 2023). Most recently, the same approach was applied to 30 (23 domestic, 7 imported) total BW brands, available commercially in the US and comprising public-TW-sourced (7 brands) and spring–sourced (23 brands) BW (Bradley et al., 2023b). Potential human–health effects of individual and aggregate POU-DW exposures have been explored based on multiple lines of evidence including cumulative effects–weighted quotients for mixtures, such as cumulative apical–benchmark-based Toxicity Quotients ($\sum_{\text{TQ}}$) (Bradley et al., 2021a; Corsi et al., 2019) and cumulative molecular-scale Exposure-Activity Ratio(s) ($\sum_{EAR}$) (Blackwell et al., 2017). However, evolving apical-effects and molecular–effects understandings and resultant changes in corresponding benchmark metrics undermine direct comparisons of human–health risks and vertebrate–effects potentials across studies and distribution pipelines. The purpose of this paper is to provide a meta-analysis (quantitative synthesis) of previous studies by this research group, directly comparing POU–DW contaminant–mixture exposures and corresponding potential human–health effects of private-TW, public-TW, and BW by briefly summarizing exposure results and harmonizing apical-health-benchmark-weighted and bioactivity–weighted effects assessments across studies. Specific hypotheses addressed herein include:

- POU-DW exposures of potential human–health concern are common to all 3 distribution pipelines, although some differences in individual contaminant and contaminant–class exposures may exist.
- POU-DW exposures and associated effects potentials vary widely within all distribution pipelines.
- Across all studies and samples, systematic differences between distribution pipelines with respect to cumulative exposures and predicted effects are limited.

## 2. Methods

### 2.1. Sample collection and target analyses

For private-TW and public-TW samples, untreated kitchen cold-water taps were sampled at the participants’ convenience throughout the day without pre-cleaning, screen removal, or Lead and Copper Rule (U.S. Environmental Protection Agency 2008, 2021) stagnant-sample protocols, as described (Romanok et al., 2018). For BW, organic–chemical samples were prepared by pouring water from the original packaging into the appropriate analytical sample bottle (Bradley et al., 2023b). Controls for sampling artifacts (field blanks) were prepared in the same manner in the field (private TW, public TW) or in the laboratory (BW) with reagent blank waters (Bradley et al., 2023b). For inorganic–chemical and microbial analyses, BW samples were delivered in their original packaging to the analytical laboratory for processing and analysis (Bradley et al., 2023b). Collected samples were shipped the day of collection on ice overnight to respective analytical laboratories.

Briefly, private–TW, public–TW, and BW samples were analyzed using the same 3 inorganic (35 analytes), 8 target-organic (465 unique analytes), 3 field parameter, and 11 microbial–indicator methods, as discussed (Bradley et al., 2018; Bradley et al., 2020; Bradley et al., 2021a; Romanok et al., 2018) and described in detail previously (American Public Health Association 2018a, b, c, d; Ball and McCleskey 2003; Barringer and Johnsson 1996; Cohn et al., 1968; Furlong et al., 2014; Graham et al., 2010; Hajna 1955; Hergenreder 2011; Hladik et al., 2014; Hoffman et al., 1996; Kolpin et al., 2021; Kozak et al., 2013; Levin and Cabelli 1972; Lilley and Brewer 1953; Lisle and Priscu 2004; Loftin et al., 2016; McCleskey et al., 2019; Petrisek and Hall 2017; Rose et al., 2016; Sandstrom et al., 2015; U.S. Environmental Protection Agency 1997, 2014; U.S. Geological Survey variously dated). Pharmaceutical and pesticide samples were syringe filtered (0.7 μm nominal pore size, glass fiber) in the field (TW) and in the laboratory (BW) prior to shipment and laboratory analyses (Furlong et al., 2014; Sandstrom et al., 2015).

### 2.2. Data Harmonization and Statistics

Some of the studies considered in this meta-analysis incorporated additional methods and associated analytes beyond those discussed above. To ensure intercomparability of exposures and risk screening metrics, corresponding data were constrained to only the above core analytes and methods maintained across all studies (Table S1). Complete method details including reporting limits, quality assurance/quality control (QA/QC) data, and final POU-DW exposure results are available in the original Supporting Information files (Bradley et al., 2018; Bradley et al., 2020; Bradley et al., 2021a; Bradley et al., 2021b; Bradley et al., 2022; Bradley et al., 2023a; Smalling et al., 2023) and in corresponding, publicly–accessible, machine-readable, ScienceBase data releases at (Meppelink et al., 2023; Romanok and Bradley 2018; Romanok et al., 2019; Romanok et al., 2020; Romanok and Bradley, 2021a, b; Romanok et al., 2021; Romanok et al., 2022; Romanok et al., 2023). Herein, differences (data centroids and dispersions) between private–TW, public–TW, and BW sample groups were assessed by nonparametric One–way PERMANOVA (n = 9999 permutations) on Euclidean distance (Paleontological Statistics, PAST, vers. 4) (Hammer et al., 2001).

### 2.3. Individual contaminant Human-Health–Benchmark screening

POU-DW exposures to individual contaminants were compared to enforceable EPA MCL (U.S. Environmental Protection Agency 2024g) and FDA SOQ (U.S. Food & Drug Administration 2024b) to provide regulatory context for public-supply TW and BW, respectively, and as a frame of reference for private-TW. However, because the EPA MCL values, on which FDA SOQ are based, take technical feasibility and cost into consideration (U.S. Environmental Protection Agency 2024e), the potential for apical human-health effects of individual contaminant exposures was screened based on the MCL goal(s) (MCLG), “the maximum level of a contaminant in drinking water at which no known or anticipated adverse effect on the health of persons would occur, allowing an adequate margin of safety,” when considering sensitive (infants, children, elderly, immune– or disease-compromised) sub-populations (U.S. Environmental Protection Agency 2024g), and other similar state and international DW human–health advisories.

### 2.4. Cumulative contaminant Human-Health–Benchmark Hazard Index (HI) screening

A human–health DW–benchmark–based cumulative risk screening assessment of aggregate inorganic and organic contaminant risk was conducted consistent with World Health Organization/International Programme on Chemical Safety [WHO/IPCS] framework Tier 1 Hazard Index risk screening (Meek et al., 2011), European Food Safety Authority Tier 1 Reference Point Index (RPI) risk screening (EFSA Scientific Committee et al., 2019), and 2023 EPA (U.S. Environmental Protection Agency 2023b) guidance, as described previously (e.g., Bradley et al., 2023a; Bradley et al., 2023b). The toxEval version 1.3.0 package (De Cicco et al., 2018) of the open source statistical software R (R Development Core Team 2019) was used to sum (non–interactive concentration addition model (e.g., Altenburger et al., 2018; Cedergreen et al., 2008; Stalter et al., 2020)) the TQ (ratio of detected concentration to corresponding health–based DW benchmark) of individual detections to estimate sample-specific cumulative TQ ($\sum_{\text{TQ}}$) (Corsi et al., 2019). For each detected analyte, the most protective human–health DW benchmark (i.e., lowest benchmark concentration) among the following was employed: NPDWR MCLG (U.S. Environmental Protection Agency 2024d, e), EPA Drinking-Water Health Advisories (DWHA)(U.S. Environmental Protection Agency 2018), WHO guidelines (World Health Organization (WHO) 2011), state MCL or DWHA (e.g., Minnesota Department of Health 2023), or USGS Health-Based Screening Level (HBSL) or Human Health Benchmarks for Pesticides (HHBP) (Norman et al., 2018)). MCLG are set at “zero” for drinking-water contaminants (e.g., bromodichloromethane, lead [Pb]), which “may cause cancer” and for which “there is no dose below which the chemical is considered safe,” including for sensitive (infants, children, elderly, immune– or disease-compromised) sub-populations (U.S. Environmental Protection Agency 2024d, e, g). For the $\sum_{\text{TQ}}$ assessment employed herein, MCLG values of “zero” were set to 0.1 μg L^−1^ for metals (arsenic [As], lead [Pb], uranium [U]), DBP, and VOC and to 0.0001 μg L^−1^ for perfluorooctane sulfonate (PFOS) and perfluorooctanoic acid (PFOA). $\sum_{\text{TQ}}$ results and respective health–based benchmarks are summarized in Tables S2a–S2b.

### 2.5. Cumulative contaminant Molecular–Effects screening

Potential molecular–level effects of mixed-organic contaminant exposures also were explored, using two exposure-activity ratio (EAR) approaches based on Toxicity ForeCaster (ToxCast) (Filer et al., 2017) high-throughput data (U.S. Environmental Protection Agency 2024b). In contrast to the human–health DW–concentration benchmarks employed in the mathematically–analogous $\sum_{\text{TQ}}$ assessment above, chemical–specific ToxCast metrics are *in vitro* estimates of exposure–response relations at the site of molecular activity. The first EAR approach, employed in the previous POU–DW studies (Bradley et al., 2018; Bradley et al., 2020; Bradley et al., 2021a; Bradley et al., 2021b; Bradley et al., 2022; Bradley et al., 2023a; Bradley et al., 2023b; Smalling et al., 2023) and emphasized herein, assumes that the measured DW exposure provides a reasonable first–level estimate of the *in vivo* molecular-level exposure. Accordingly, the R package (R Development Core Team 2019) toxEval version 1.3.0 (De Cicco et al., 2018) was employed to sum (non–interactive concentration addition model (e.g., Altenburger et al., 2018; Cedergreen et al., 2008; Stalter et al., 2020)) individual–contaminant EAR (ratio of the detected contaminant concentration to the contaminant-specific “activity concentration at cutoff” for a positive response (ACC) metric from ToxCast (U.S. Environmental Protection Agency 2024b)) to estimate sample–specific cumulative EAR ($\sum_{\text{EAR}}$) (Blackwell et al., 2017; Bradley et al., 2018; Bradley et al., 2020). ACC data in the toxEval v1.3.0 employed in the present study were from the November 2022 invitroDBv3.5 release of the ToxCast database (U.S. Environmental Protection Agency National Center for Computational Toxicology 2023). Non-specific-endpoint, baseline, and unreliable response–curve assays were excluded (Blackwell et al., 2017; Bradley et al., 2018; Bradley et al., 2020). $\sum_{\text{EAR}}$ results and exclusions are summarized in Tables S3a–S3c. Note, a previous study, which applied both ($\sum_{\text{EAR}}$ and $\sum_{\text{TQ}}$) approaches to organic environmental contaminants, reported approximate contaminant-specific equivalency of the widely–employed TQ = 0.1 screening–level threshold of concern and EAR = 0.001 (Corsi et al., 2019). Accordingly, EAR (and $\sum_{\text{EAR}}$) = 0.001 was employed herein as a screening level of potential concern (i.e., for additional investigation and characterization) but not as a direct indicator of health risk, due to uncertainties in *in vitro* to *in vivo* extrapolation (El-Masri et al., 2022; Villeneuve et al., 2019) and the fact that measured bioactivities are not necessarily adverse and may, in some cases, reflect adaptive (e.g., activation of xenobiotic metabolism (Hakkola et al., 2018)) responses.

Secondly, to aid in translation of a ToxCast effect concentration (i.e., ACC reported as a water concentration) to a human–consumption–relevant exposure metric, contaminant–specific EAR also were calculated based on measured equivalent dose (MED) estimates and the AED_95_ estimates derived from high–throughput toxicokinetic (HTTK) modeling using the data and models in the R package *httk* version 2.0.3 (Pearce et al., 2017), as described (Paul Friedman et al., 2020). EAR_MED_ is described by the following equation:

$$EAR_{MED}=\frac{MED}{AED95}$$

Where MED (mg kg^−1^ d^−1^) was calculated from the measured POU–DW concentration (μg L^−1^), based on published guidelines (U.S. Environmental Protection Agency 2019) and the most conservative assumption (for birth to < 1 month) of 200 mg kg^−1^ d^−1^ DW ingestion.


$$\text{MED}=\frac{\text{μg substance}}{\text{L}}*\frac{0.2\;\text{L water consumed}}{\text{kg}*\text{d}}*\frac{0.001\;\text{mg}}{\text{μg}}$$

The 5th percentile administered equivalent dose (AED) estimate for the population (AED_95_; mg kg^−1^ d^−1^) was calculated using the calc_mc_oral_equiv() function in the *httk* R package, the 95th quantile for the steady-state concentration (Css) in the plasma (Css_95_), restrictive clearance, the 3–compartment steady-state model, and assuming 100 % bioavailability. The 3-compartment steady-state model estimates the Css and requires chemical-specific information on plasma protein binding and hepatic intrinsic clearance, which is available in the *httk* package.


$${\text{AED}}_{95}=\text{bioactive concentration}\;(\text{μM})*\frac{1\frac{\text{mg}}{\text{kg}}/\text{day}}{{\text{CsS}}_{95}(\text{μM})}$$

To approximate population variability, Monte Carlo simulation was used to vary the following toxicokinetic parameters: first-order hepatic metabolic clearance, plasma protein binding, liver blood flow, and the rate of clearance via the kidney (Pearce et al., 2017; Wetmore et al., 2012; Wetmore et al., 2014). AED_95_ values were calculated using ACC values from the November 2022 invitroDBv3.5 release of the ToxCast database (U.S. Environmental Protection Agency National Center for Computational Toxicology 2023) and the EAR_MED_ value for each detected chemical was calculated using the 5th percentile of all AED_95_ for that chemical. $\sum_{\text{EAR-MED}}$ results (same exclusions as for $\sum_{\text{EAR}}$, Table S3a) are summarized in Tables S3d.

## 3. Results and discussion

### 3.1. Meta-Analysis of target analyte exposures

**Fig. 1.**
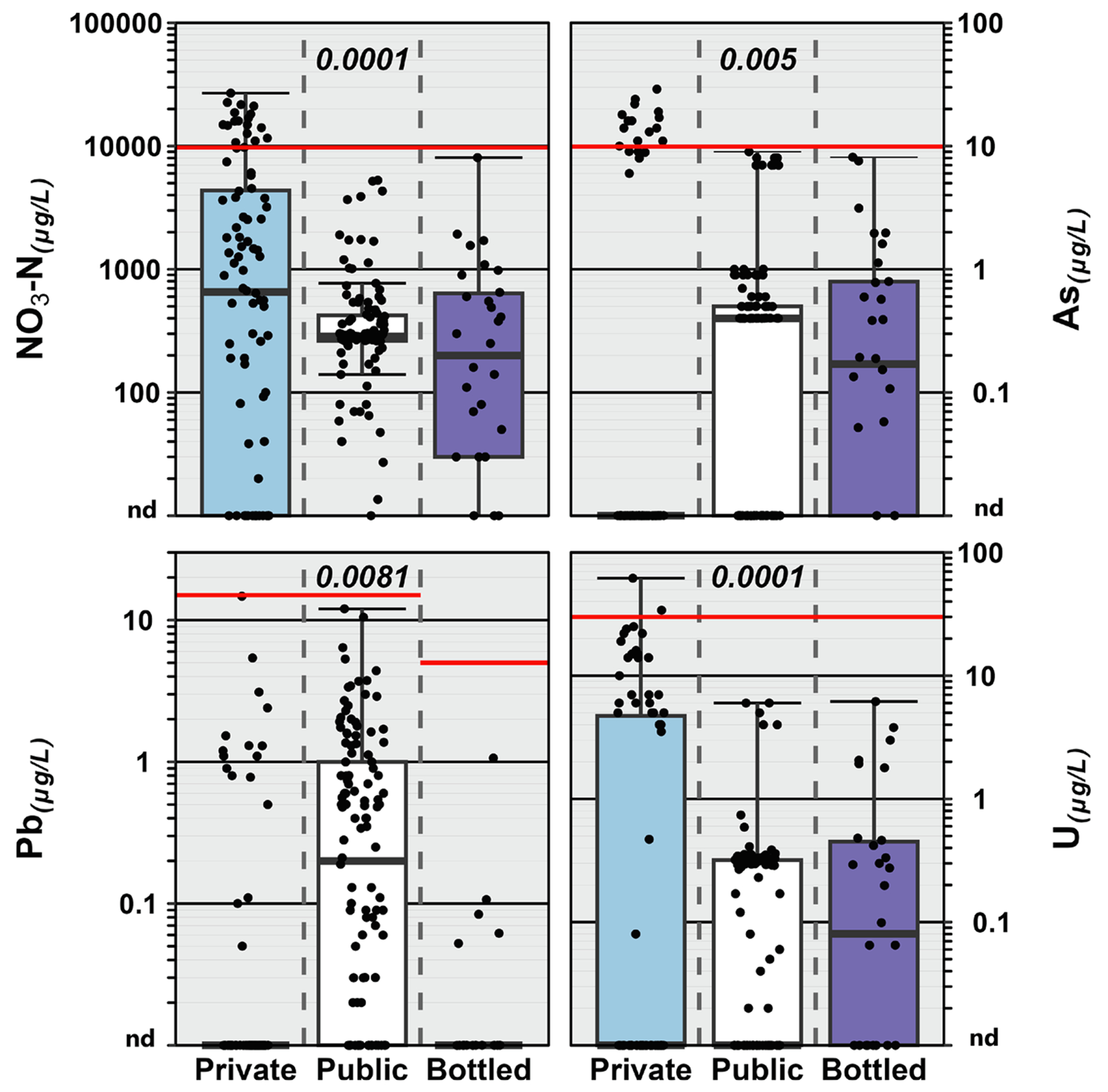
Group comparison of concentrations of select inorganics detected in private–tapwater (Private; cyan), public–tapwater (Public; white), and bottled–water (Bottled; purple) samples. Solid red lines indicate Environmental Protection Agency (EPA) maximum contaminant level (MCL: enforceable for public tapwater; reference only for private tapwater) and Food and Drug Administration (FDA) standard of quality (SOQ: enforceable for bottled water). Maximum contaminant level goals (MCLG) for As, U, and Pb are zero. For NO_3_-N, MCLG and MCL are the same. Circles (●) are data for individual samples. Boxes, centerlines, and whiskers indicate interquartile range, median, and 5th and 95th percentiles, respectively. Numbers at top center of plots indicate the permuted probability that the centroids and dispersions are the same (PERMANOVA; 9999 permutations) across all drinking-water types. (For interpretation of the references to colour in this figure legend, the reader is referred to the web version of this article.)

**Fig. 2.**
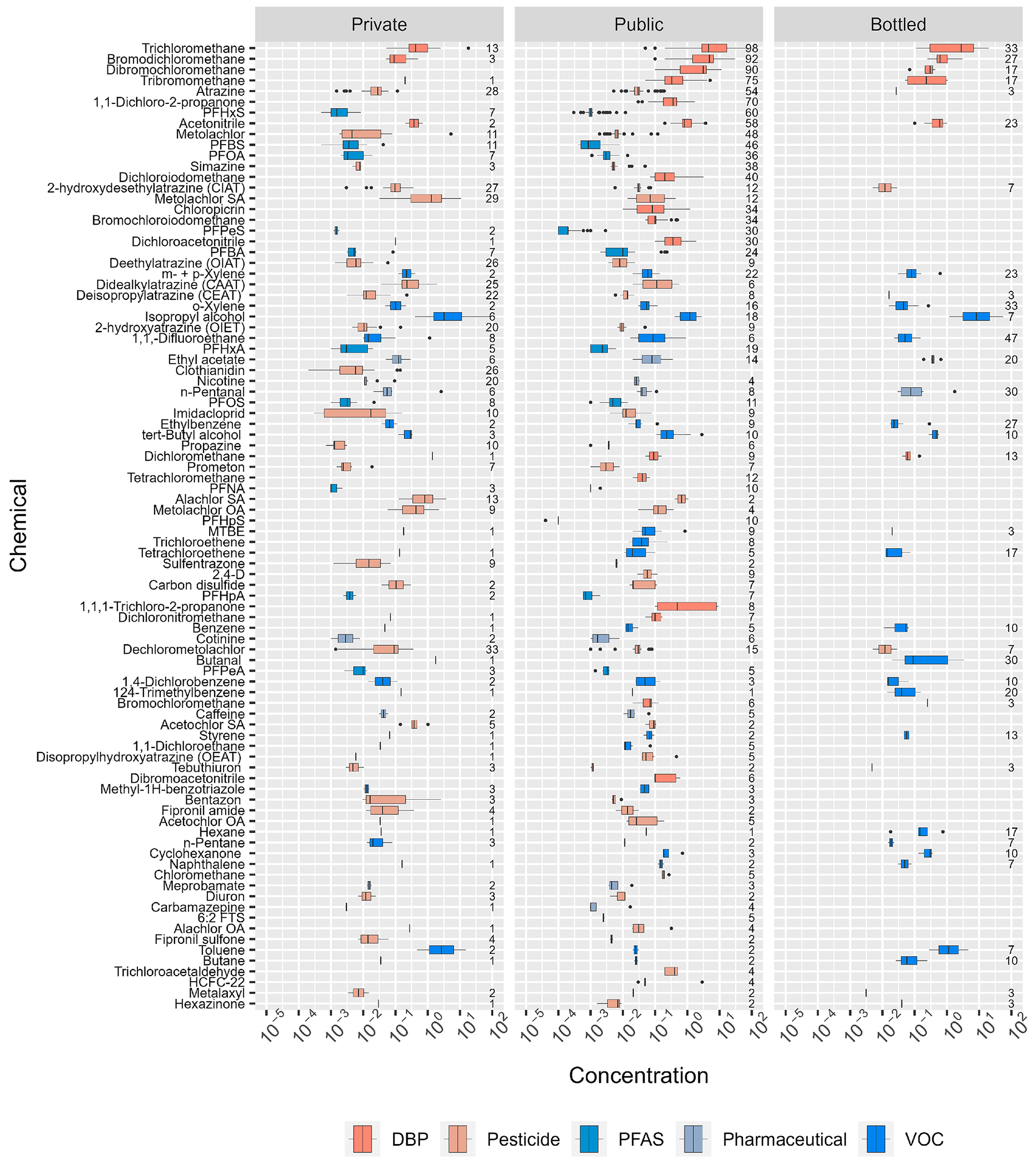
Detected concentrations (circles, μg/L) of organics in private–tapwater (Private; left), public–tapwater (Public; center), and bottled–water (Bottled; right), arranged top to bottom in order of decreasing overall detection frequency. Circles (●) are data for individual samples. Boxes, centerlines, and whiskers indicate interquartile range, median, and 5th and 95th percentiles, respectively. Box colors identify disinfection byproduct (DBP), pesticide, per/polyfluoroalkyl substances (PFAS), pharmaceutical, and volatile organic chemical (VOC) classes, as shown in legend. Numbers on right Y-axis indicate compound-/supply-specific detection frequencies (percent of samples).

**Fig. 3.**
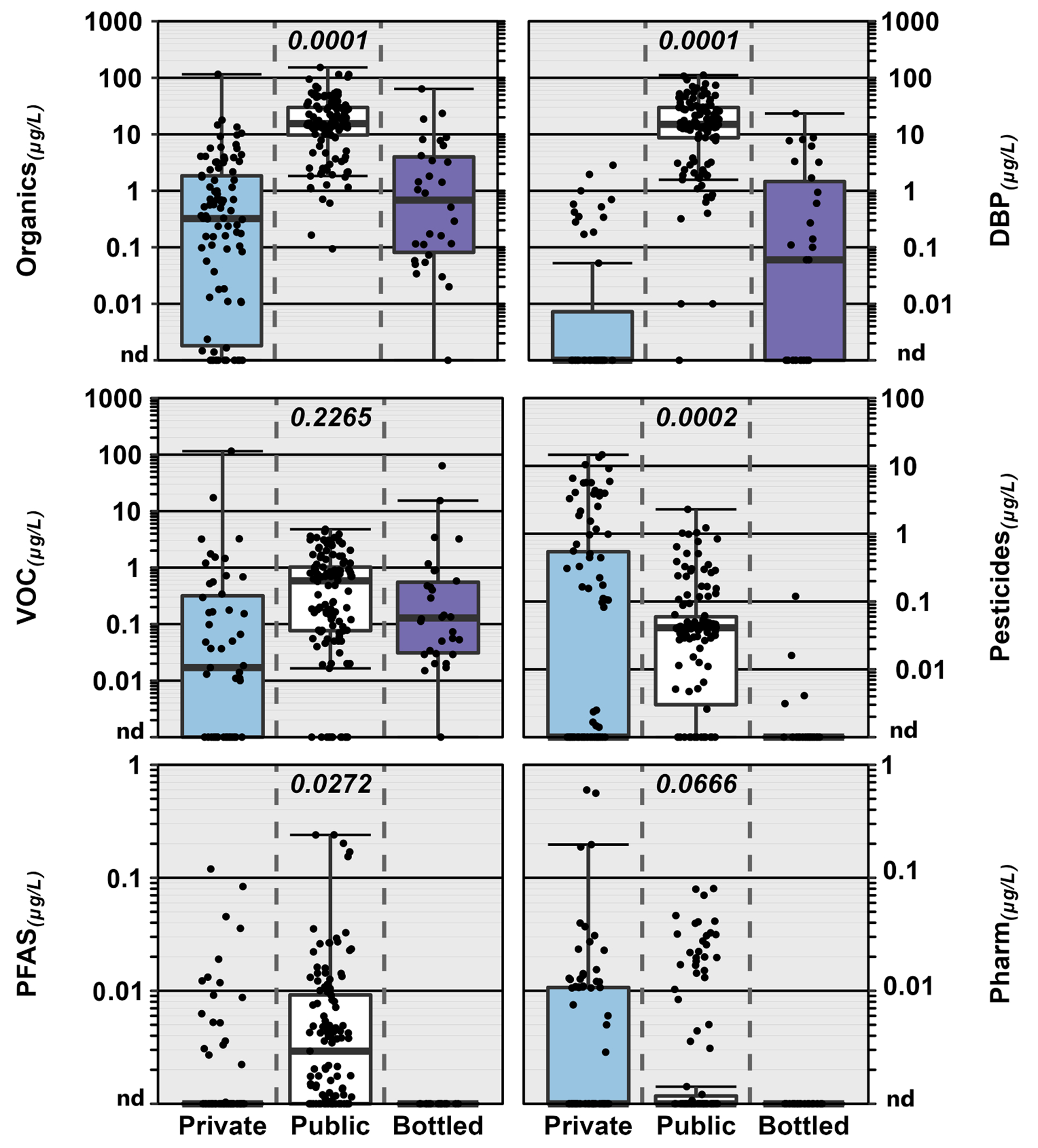
Group comparisons of cumulative concentrations (circles, μg/L) of all organics (upper left) and of select organic classes detected in private–tapwater (Private; cyan fill), public–tapwater (Public; white), and bottled–water (Bottled; purple) samples. DBP, VOC, and PFAS are disinfection byproducts, volatile organic chemicals, and per/polyfluoroalkyl substances, respectively. For DBP, the solid red line indicates the Environmental Protection Agency (EPA) maximum contaminant level (MCL: enforceable for public tapwater; reference only for private tapwater) and Food and Drug Administration (FDA) standard of quality (SOQ: enforceable for bottled water) for trihalomethanes (80 μg/L). For PFAS, the solid red line indicates the Environmental Protection Agency (EPA) maximum contaminant level (MCL: enforceable for public tapwater; reference only for private tapwater) and presumptive Food and Drug Administration (FDA) standard of quality (SOQ: enforceable for bottled water) for PFOS or PFOA (0.004 μg/L). Circles (●) are data for individual samples. Boxes, centerlines, and whiskers indicate interquartile range, median, and 5th and 95th percentiles, respectively. Numbers at top center of plots indicate the permuted probability that the centroids and dispersions are the same (PERMANOVA; 9999 permutations) across all drinking-water types. (For interpretation of the references to colour in this figure legend, the reader is referred to the web version of this article.)

**Fig. 4.**
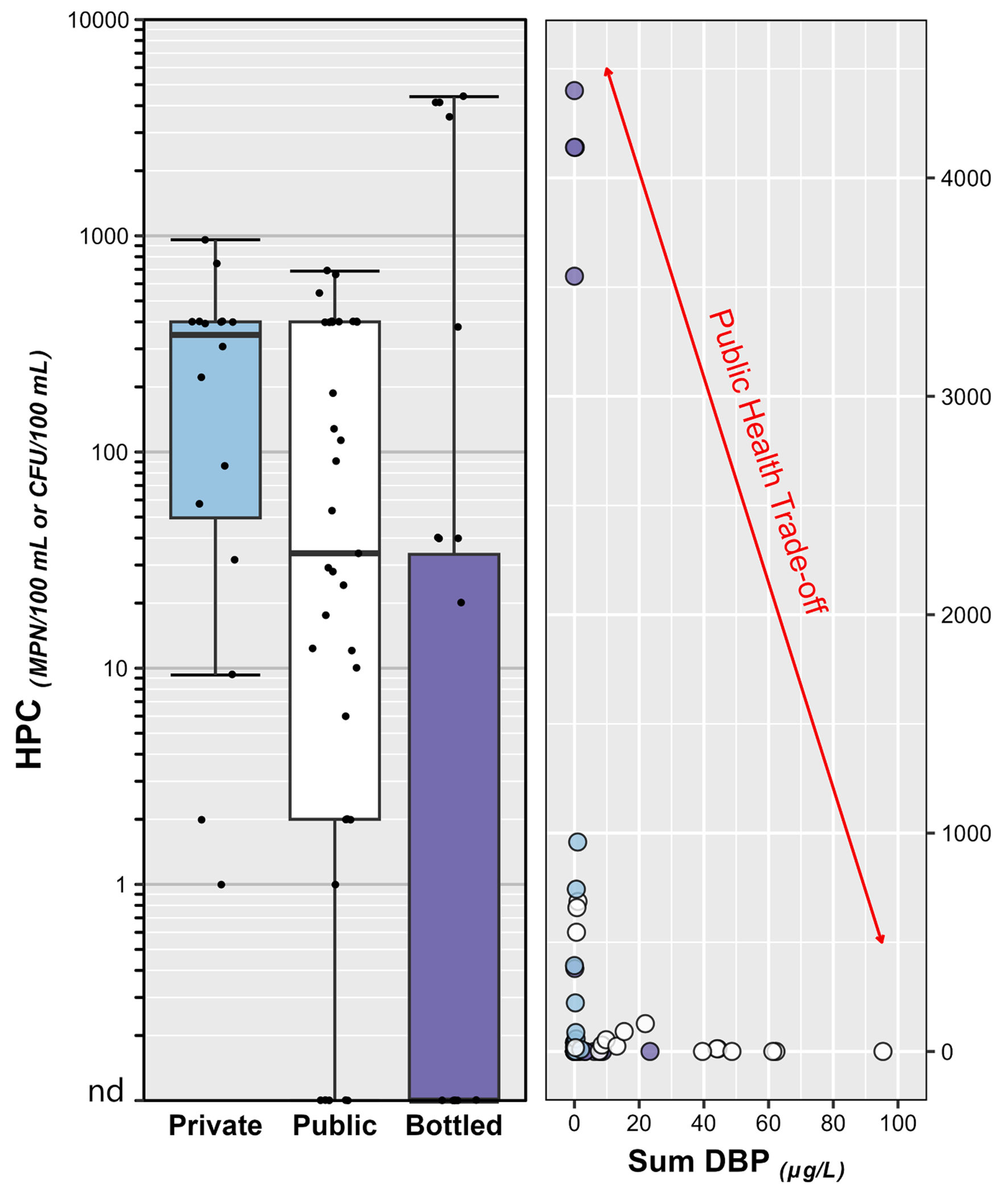
**Left Plot** – Group comparisons of heterotrophic plate count (HPC) results, a general indicator of concentrations (colony forming units [CFU] per 100 mL) of viable heterotrophic microorganisms in private–tapwater (Private; cyan fill), public–tapwater (Public; white), and bottled–water (Bottled; purple) samples. Circles (●) are data for individual samples. Boxes, centerlines, and whiskers indicate interquartile range, median, and 5th and 95th percentiles, respectively. **Right Plot** – Scatterplot of HPC versus cumulative concentration of disinfection byproducts (DBP, μg/L) in private–tapwater (Private; cyan fill), public–tapwater (Public; white), and bottled–water (Bottled; purple) samples, illustrating the public-health trade-off of chlorine disinfection of drinking water. (For interpretation of the references to colour in this figure legend, the reader is referred to the web version of this article.)

In aggregate, broad-scope target-analyte exposure results published to date by this group included 254 POU-DW (98 private–TW, 126 public–TW, 30 BW) samples (Bradley et al., 2018; Bradley et al., 2020; Bradley et al., 2021a; Bradley et al., 2021b; Bradley et al., 2022; Bradley et al., 2023a; Bradley et al., 2023b; Smalling et al., 2023); select data are summarized by DW supply in Figs. 1–4, S1–S2. Consistent with an increasingly anthropized water cycle and with Hypothesis I, multiple regulated and unregulated chemical (inorganic, organic) and microbial analytes were routinely detected in samples from all three DW supplies, with 2 or more detections of potential human–health concern observed in 90 % of samples (Table S2b). Of the 465 unique organics analyzed across all studies, 184 (40 %) were detected at least once, with compound-specific detection frequencies up to 57 % across all studies and up to 29 %, 98 %, and 47 % in private-TW, public-TW, and BW, respectively. Consistent with Hypotheses II and III, POU-DW exposures varied widely within all three DW supplies, with considerable overlap in detected concentrations and little systematic difference when considering cumulative inorganic/organic chemical and microbial exposures. The results illustrate the importance of balanced, intercomparable POU–DW contaminant exposure information across all three DW supplies to support objective consumer decision–making, including increasingly crucial public engagement in 1) source-water protection, 2) water conservation and re-use, 3) long–term sustainable TW–treatment improvements at the source, point–of–entry, and point–of–use, and 4) minimizing high energy, cost, and environmental–waste choices, like single-use individual BW consumption, under non–emergency situations.

### 3.2. Individual contaminant risk screening

Notably, no exceedance of enforceable MCL or SOQ standards for inorganics were observed in public–TW or BW, respectively, but greater than MCL (frame of reference; not enforceable in federally–unregulated private-TW) concentrations of inorganic contaminants were observed in private-TW (Fig. 1). The lack of exceedances in regulated public–TW and BW is consistent with broadly effective regulatory monitoring and treatment. In contrast, greater than MCL concentrations in private-well samples reiterate the inherent risks of unrecognized contaminant exposures in federally–unregulated and widely–unmonitored private–TW and illustrate the potential benefits of systematic monitoring of private-TW (Zheng and Flanagan 2017), with an analytical scope that realistically reflects the documented complexity of environmental contamination (Bradley et al., 2017; Glassmeyer et al., 2017; Moschet et al., 2014; Schaider et al., 2014; Schaider et al., 2016), to mitigate unrecognized, adverse human-health exposures. As noted, enforceable EPA MCL (U.S. Environmental Protection Agency 2024g) and FDA SOQ (U.S. Food & Drug Administration 2024b) are presented for regulatory context, but emphasis here and in the below risk screening discussion is placed on MCLG that identify a maximum level of a contaminant in drinking water at which no known or anticipated adverse effect on the health of persons would occur, allowing an adequate margin of safety, when considering sensitive (infants, children, elderly, immune– or disease–compromised) sub-populations (U.S. Environmental Protection Agency 2024g) and other DW human–health-only advisories.

Consistent with Hypothesis I, greater than MCLG concentrations were observed for multiple inorganics across all three DW supplies. Arsenic (As), lead (Pb), and uranium (U) have MCLG of “zero” (U.S. Environmental Protection Agency 2024e). Redox-reactive, geogenic (i.e., geologically derived) As was detected in 21 %, 51 %, and 67 % of private-TW, public-TW, and BW samples, respectively, with concentrations greater than 1 μg L^−1^ in 21 %, 10 %, and 23 %, respectively; the lower detection frequency for ≥ 1 μg L^−1^ As in public-TW is consistent with effective regulatory–compliance monitoring and treatment and with substantial (86 % of public–TW samples herein) surface–water (typically low dissolved As concentrations) sourcing, in contrast to entirely and predominantly (77 % of BW samples herein) groundwater-based private–TW and BW, respectively. Pb was detected in 17 %, 67 %, and 17 % of private–TW, public–TW, and BW samples, respectively, with concentrations greater than 1 μg L^−1^ in 10 %, 25 %, and 3 %, respectively; note, more than half of the public-TW Pb detections (including at concentrations greater than 1 μg L^−1^) were POU–DW samples from the greater Chicago area (Bradley et al., 2020), which has documented service-line Pb issues (Del Toral et al., 2013). Detections of Pb in BW samples are particularly noteworthy because occurrence in DW is primarily attributed to legacy use in distribution–system and premise–plumbing infrastructure (Triantafyllidou and Edwards 2012b) and, thus, unexpected in modern BW–packaging facilities. Redox-reactive, geogenic U was detected in 31 %, 50 %, and 57 % of private–TW, public–TW, and BW samples, respectively, with concentrations greater than 1 μg L^−1^ in 29 %, 4 %, and 20 %, respectively. Manganese (Mn); a DW contaminant of emerging concern (Iyare 2019; Ramachandran et al., 2021) for cognitive, neurodevelopmental, and behavioral effects of long–term exposures in children and bottle-fed infants; was detected in all three DW supplies. No MCL, MCLG, or SOQ currently exist for Mn, but USEPA maintains a 300 μg L^−1^ life–time DWHA (U.S. Environmental Protection Agency 2018) and the Minnesota Department of Health established a health-based value of 100 μg L^−1^ (Table S2a). The latter was exceeded in 14 % of private–TW and 8 % of public–TW samples, but not in any tested BW. Median concentrations (<0.2 mg L^−1^) of fluoride in samples from all three DW supplies were below the 0.7 mg L^−1^ US Public Health Service (2015) recommended DW concentration to prevent childhood dental caries (Fig. S1), in line with previous concerns for the dental health of children (Cochrane et al., 2006; Horowitz et al., 2015; Mills et al., 2010). While caution is warranted in interpreting the above detection-frequency results due to known contaminant issues in some of these community–based/community-driven studies (e.g., As and U in the Northern Plains Nations (Bradley et al., 2022); Pb in the Chicago area (Bradley et al., 2020)), the results clearly demonstrate that exposures to inorganic contaminants at levels of concern for adverse human–health effects to vulnerable sub-populations occur in all three DW supplies.

Prior to release of the EPA 2024 final PFAS National Primary Drinking Water Rule (EPA PFAS Rule)(U.S. Environmental Protection Agency 2024f), concentrations greater than MCL/SOQ (Figs. 3) were not observed for organics in private-TW or BW and were rarely observed (2 %) in public-TW (80 μg L^−1^ trihalomethane (THM) MCL exceeded in three samples in Puerto Rico post-Hurricane Maria infrastructure (Bradley et al., 2021b)). This general lack of exceedances in regulated public-TW and BW was consistent with effective regulatory compliance monitoring and treatment, as noted above, and, importantly, with the fact that many target analytes, including PFAS, were not federally regulated (U.S. Environmental Protection Agency 2024e). The fact, that concentrations in 10 % of public–TW (4 % of private–TW) samples would equal or exceed EPA PFAS Rule MCL levels (compliance 5 years after Federal Register publication (U.S. Environmental Protection Agency 2024c)), illustrates the challenge that the PFAS class poses to DW regulation, monitoring, and treatment.

Consistent with Hypothesis I, multiple organics with MCLG of “zero” were observed across all three DW-supplies. Among these, six industrial, volatile organic chemicals (VOC; namely tetrachloroethene [PCE], trichloroethene [TCE], vinyl chloride, tetrachloromethane, benzene, 1,2–dichloropropane) were detected at frequencies up to 2 %, 10 %, and 16 % in private–TW, public–TW, and BW samples, respectively. Also, among these, three VOC (bromodichloromethane, tribromomethane, dichloromethane) associated with chlorine–disinfected public-TW (i.e., DBP) were detected in private–TW, public–TW, and BW samples at frequencies up to 1 %, 84 %, and 27 % (100 % of public–TW–sourced BW samples), respectively. The EPA PFAS Rule established, among others, MCLG of “zero” and MCL = 4 ng L^−1^ for perfluorooctanesulfonic acid (PFOS) and perfluorooctanoic acid (PFOA) and MCLG/MCL = 10 ng L^−1^ for perfluorohexansulfonate (PFHxS) and perfluorononanoic acid (PFNA) (U.S. Environmental Protection Agency 2024c, f); at least one of which was detected (greater than MCLG = “zero” for PFOS and PFOA) in up to 7 % of private–TW and 26 % of public–TW samples but not in BW. Note, PFAS have been reported widely in BW (Akhbarizadeh et al., 2020; Chow et al., 2021; Gellrich et al., 2013; Schwanz et al., 2016; Teymoorian et al., 2023), albeit generally at concentrations below the detection limits of the Bradley et al. (2023a) study. As for the inorganics above, caution is warranted in interpreting the detection–frequency results, due to known contaminant issues in some of these community–based/community–driven studies (e.g., PFAS in Cape Cod (Bradley et al., 2021a)). Nevertheless, these results clearly demonstrate that organic–contaminant exposures of concern for adverse human–health effects to vulnerable sub–populations also occur in all three DW–supplies, emphasizing the importance of continued monitoring of POU-DW with an analytical scope that realistically reflects the documented complexity of environmental organic contamination (Bradley et al., 2017; Glassmeyer et al., 2017; Moschet et al., 2014; Schaider et al., 2014; Schaider et al., 2016), to mitigate unrecognized, adverse human-health exposures.

Viable bacteria including potential pathogens were assessed in select POU-DW studies. General heterotrophs (heterotrophic plate counts, HPC) were commonly detected across all three POU-DW supplies (Fig. 4). HPC bacteria; which are common in the environment, routinely detected in DW, and not intrinsic health concerns; are useful indicators of source-water quality, system maintenance, disinfection efficacy, and post-treatment regrowth in the distribution “pipeline” prior to consumption (U.S. Environmental Protection Agency 2024e). Surprisingly, the highest HPC results in these studies were observed in BW samples. While two of the highest HPC results were observed in nominally, spring-sourced BW with no listed filtration or treatment, comparable results in nominally ozone–/UV–disinfected BW illustrate concerns for potential biological–regrowth in the absence of residual disinfectant. The clear inverse relation between HPC and DBP residuals is shown in Fig. 4. Growth on putative pathogen selective media (e.g., *Legionella* spp., *Staphylococci*) was observed sporadically in POU-DW samples from all three supplies, albeit generally at or near detection-limit levels.

Notable differences in exposures, with important implications for POU-DW quality improvements, also were observed between distribution pipelines for individual contaminants or contaminant classes and were attributable in part to respective differences in drinking water regulation and compliance monitoring. Critically, as noted above, greater than MCL or AL (frame of reference; MCL/AL not enforceable in federally–unregulated private-TW) inorganic (As, Cu, NO_3_–N, Pb, U) and, with the EPA PFAS Rule, organic (PFOS, PFOA, PFHxS) concentrations were observed in private–TW, emphasizing the elevated risk of unrecognized adverse exposure in generally unregulated and unmonitored private supplies and the potential benefits of broader monitoring of private-well water quality (Zheng and Flanagan 2017). Unsurprisingly, DBP dominated detections and cumulative concentrations of organics in public–TW, due to common–place chlorine-based disinfection of public supplies in the US. Remarkably, DBP residuals of chlorine disinfection (e.g., trichloromethane, bromodichloromethane, acetonitrile, tribromomethane) also were detected frequently (53 %) in BW, in every purified-TW BW sample but also in some nominally untreated (i. e., no reported chlorine disinfection) spring-sourced BW. Public–TW is the primary source water for single–serving BW in the US (Buchwald et al., 2023; Corson-Dosch et al., 2023; Food and Water Watch.org 2018) and DBP have been reported previously in purportedly untreated spring-sourced BW samples (Stanhope et al., 2020). The infectious–disease prevention benefits of chemical disinfection prior to distribution and of residual disinfectant at the tap are well–documented (Reynolds et al., 2008; Richardson et al., 2007; Schoenen 2002) and supported by the clear inverse relation between HPC and DBP in Fig. 4. Acknowledging the smaller number of BW samples assessed (30 versus 98 private-TW and 126 public-TW), no pharmaceutical or PFAS contaminants were detected in BW samples by this group (Bradley et al., 2023b); however, several previous BW studies have reported pharmaceuticals (Akhbarizadeh et al., 2020; Lardy-Fontan et al., 2017; Wang et al., 2021) and PFAS (Akhbarizadeh et al., 2020; Chow et al., 2021; Gellrich et al., 2013; Schwanz et al., 2016; Teymoorian et al., 2023), the latter including greater than MCL (by law (21 U.S.C. § 349 1996), FDA must promulgate a BW SOQ that is no less protective than the EPA MCL or make a finding that such regulation is not necessary no later than 180 days before the MCL effective date; FDA SOQ for PFAS in BW have not been established at this time) concentrations (Kunacheva et al., 2010; Le Coadou et al., 2017; Schwanz et al., 2016).

### 3.3. Human-Health–Benchmark $\sum_{\text{TQ}}$ screening

Co-occurring contaminant exposures in samples from all three DW supplies, including concentrations greater than multiple health benchmarks in the same sample, demonstrate the importance of screening the cumulative risk of POU-DW organic/inorganic mixture exposures. Sample-specific cumulative toxicity quotients ($\sum_{\text{TQ}}$) were calculated for the first time for the original national pilot (Bradley et al., 2018) and greater Chicago area (Bradley et al., 2020) studies and recalculated for the remaining studies (Bradley et al., 2020; Bradley et al., 2021a; Bradley et al., 2021b; Bradley et al., 2022; Bradley et al., 2023a; Bradley et al., 2023b; Smalling et al., 2023), using a list of human-health DW benchmarks (Table S2a), updated to 1) include additional detected compounds, 2) reflect recent changes in human–health advisories, and 3) harmonize the handling of MCLG of “zero” in compound–specific TQ estimates. Regarding the second, notable recent changes include promulgation of EPA MCLG of “zero” for PFOS and PFOA (U.S. Environmental Protection Agency 2024c, f, g). Regarding the latter, in previous reports by this group, TQ denominators for contaminants with MCLG of “zero” were set to the study–specific reporting limits for the respective analytical methods, resulting in some study–to–study variability in estimated risks that was independent of observed exposures. Herein, MCLG values of “zero” were set to 0.1 μg L^−1^ for metals (As, Pb, U), DBP (bromodichloromethane, dichloromethane, tribromomethane), and VOC (benzene, tetrachloroethene, tetrachloromethane, trichloroethene, vinyl chloride) and to 0.0001 μg L^−1^ for PFOS and PFOA, a routinely–achievable analytical reporting limit for the former and an analytically–practical compromise for the latter.

**Fig. 5.**
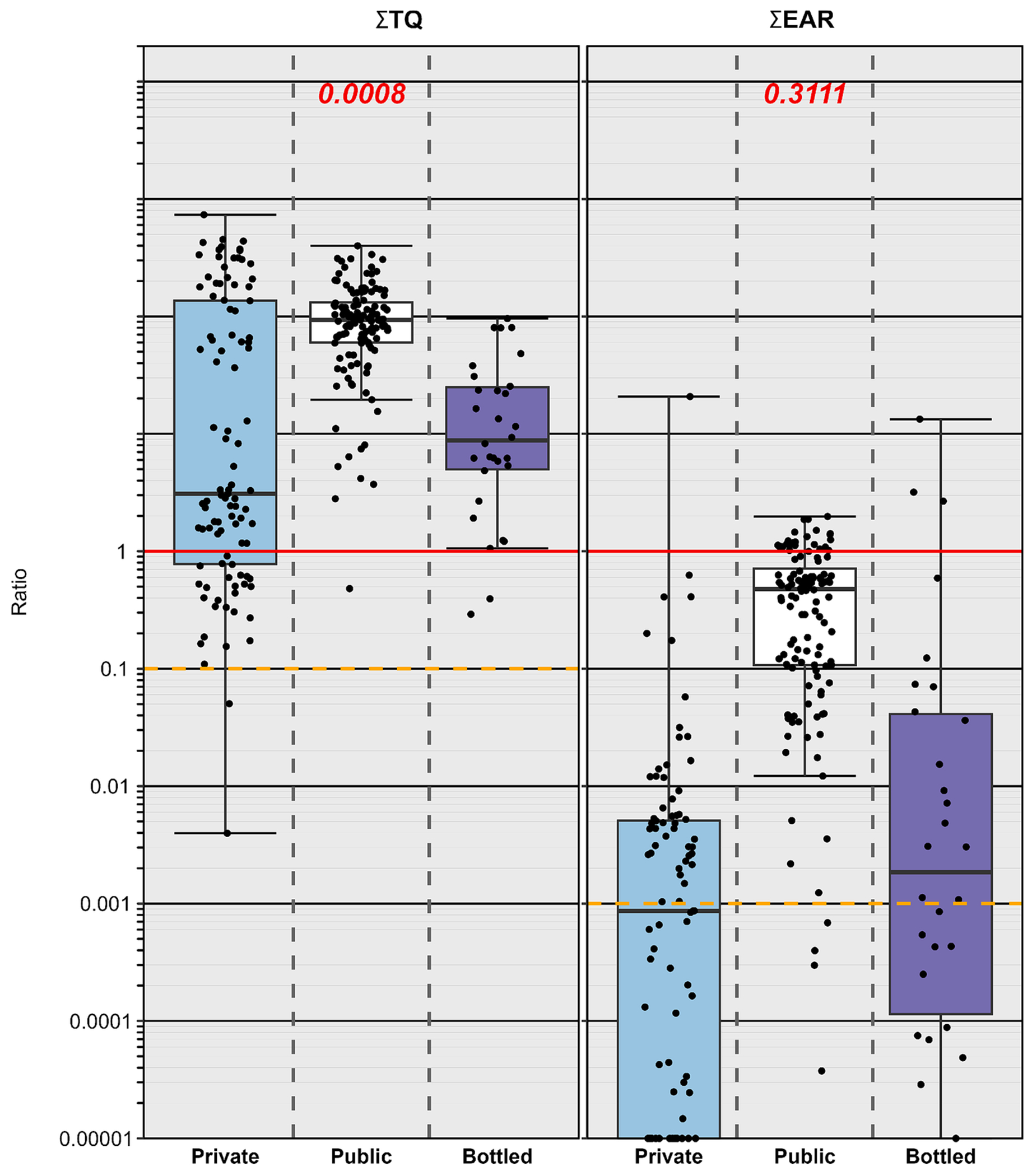
Cumulative TQ ($\sum_{\text{TQ}}$, left) and EAR ($\sum_{\text{EAR}}$, right) for analytes detected in private–tapwater (Private; cyan fill), public–tapwater (Public; white), and bottled–water (Bottled; purple) samples. Circles (●) are data for individual samples. For $\sum_{\text{TQ}}$ (left), solid red and dashed orange lines, respectively, indicate benchmark equivalent concentrations and risk–screening level (TQ = 0.1) below which little to no risk expected. For $\sum_{\text{EAR}}$ (right), solid red and dashed orange lines indicate concentrations shown to modulate effects *in vitro* and effects–screening–level (EAR = 0.001), respectively. Boxes, centerlines, and whiskers indicate interquartile range, median, and 5th and 95th percentiles, respectively. For each plot, number at the top indicates the permuted probability that the centroids and dispersions are the same across all three groups (PERMANOVA; 9999 permutations). (For interpretation of the references to colour in this figure legend, the reader is referred to the web version of this article.)

The $\sum_{\text{TQ}}$ assessment results across all studies by this group indicate that elevated human–health risks (i.e., $\sum_{\text{TQ}}\geq 1$) from contaminant-mixture exposures are common to and comparable in private-TW, public–TW, and BW samples (Fig. 5). Human–health benchmarks were available for 19 (56 %) of the 34 inorganics and 66 (36 %) of the 184 organics detected across all studies (Table S2a). Among these, 19 inorganics and 62 organics had at least one exposure resulting in an individual TQ ≥ 0.00001 and were included in the $\sum_{\text{TQ}}$ assessment. When considering cumulative POU-DW exposures within the 34 inorganic and 465 organic contaminant analytical space, less than 4 % of all samples exhibited $\sum_{\text{TQ}}$ below 0.1, a widely–employed screening–level threshold of concern (e.g., Corsi et al., 2019) and precautionary screening level for possible, albeit uncommon, positive interactions (synergism, potentiation) between detected contaminants (Cedergreen 2014; Martin et al., 2021) and for cumulative effects at less than benchmark concentrations (Kortenkamp 2022, 2023). Approximately 85 % of samples had at least one individual contaminant exposure with TQ > 1 indicating elevated risk of human-health effects for vulnerable populations. Median numbers of contaminants per sample with TQ > 1 were 1, 4, and 2 for private–TW, public–TW, and BW, respectively. Elevated cumulative human-health risk (i.e., $\sum_{\text{TQ}}>1$) was indicated in approximately 87 % of samples, with $\sum_{\text{TQ}}>1$ observed in about 72 % of private–TW, 97 % of public–TW, and 93 % of BW samples. While some statistical difference in $\sum_{\text{TQ}}$ was apparent between supplies (PERMANOVA; p = 0.0008), considerable overlap in $\sum_{\text{TQ}}$ was evident and, most important, $\sum_{\text{TQ}}$ exceeded 1 for most samples regardless of supply.

When considering both organic and inorganic contaminant exposures, the $\sum_{\text{TQ}}$ results indicate elevated cumulative–risk probabilities in all three DW supplies, with some differences in the apparent drivers of risk for different supplies. U, As, and Pb (in descending order) were common contributors to cumulative contaminant risk across all three DW supplies. In private-TW, $\sum_{\text{TQ}}\geq 1$ were driven primarily by these three trace elements, along with Mn and, to a lesser extent, NO_3_–N. This result is consistent with the facts that most of the private-TW samples were collected in rural and agriculturally–impacted Midwest and northern plains states where the most–detected organic co-contaminants were pesticides, which generally (excepting rodenticides) exhibit comparatively low vertebrate toxicity. As noted previously (Bradley et al., 2018; Bradley et al., 2020; Bradley et al., 2021a; Bradley et al., 2021b), these results reiterate the inherent human–health challenge of unmonitored private-well TW (DeSimone et al., 2015a; Focazio et al., 2006; MacDonald Gibson and Pieper 2017; Rogan and Brady 2009; Zheng and Flanagan 2017) and the potential importance of systematic private-supply monitoring (Zheng and Flanagan 2017) with an environmentally–relevant analytical scope (Bradley et al., 2017; Glassmeyer et al., 2017; Moschet et al., 2014; Schaider et al., 2014; Schaider et al., 2016). Although As, U, and less frequently Pb also contributed to cumulative risk, the primary cumulative–risk drivers in public–TW were organic contaminants with MCLG of “zero,” primarily DBP (bromodichloromethane, tribromomethane) and PFAS (PFOS, PFOA). For BW, $\sum_{\text{TQ}}\geq 1$ were driven primarily by As and U and by bromodichloromethane and tribromomethane, the latter two found in all purified–TW–sourced samples and some spring–sourced samples. The results indicate that elevated human–health risk from simultaneous exposures to multiple contaminants is common across all DW supplies, emphasizing the need for improved understanding of the adverse human–health implications of long-term exposures to low–level inorganic-/organic-contaminant mixtures across all three distribution pipelines (public–TW, private-TW, BW). Notably, these apical-effects risk results do not support commercial messaging of BW as an intrinsically safer POU–DW than public-TW or private-TW but do emphasize the need for improved source-water protection, monitoring and characterization, and treatment options across all three pipelines.

### 3.4. POU-DW exposure molecular-effects (EAR) screening

Bioactivity–weighted EAR and $\sum_{\text{EAR}}$ were calculated to 1) identify and prioritize primary organic–contaminant drivers of molecular–level human–relevant bioactivities from DW exposures, 2) independently validate the above apical–effects predictions, and 3) identify potential exposure hazards not addressed by existing human-health benchmarks. Two EAR screening approaches were employed. The first specifically assumes that measured POU–DW exposures provide a reasonable first–level estimate of the *in vivo* molecular-level exposure. Sample–specific EAR and $\sum_{\text{EAR}}$ were recalculated, for all studies (Bradley et al., 2018; Bradley et al., 2020; Bradley et al., 2021a; Bradley et al., 2021b; Bradley et al., 2022; Bradley et al., 2023a; Bradley et al., 2023b; Smalling et al., 2023) to date, to harmonize the results to the ACC data from the November 2022 invitroDBv3.5 ToxCast release (U.S. Environmental Protection Agency National Center for Computational Toxicology 2023) in toxEval v1.3.0. Consistent with the $\sum_{\text{TQ}}$ assessment results discussed above, median $\sum_{\text{EAR}}$ equaled or exceeded the $\sum_{\text{EAR}}=0.001$ screening-level for potential molecular–level effects across all three supplies (Fig. 5). ACC data were available for about 58 % (106) of the 184 organics detected across all studies (Table S3b); among these, 92 (50 % of detected) had at least one exposure resulting in an individual EAR ≥ 0.00001 and were included in the $\sum_{\text{EAR}}$ assessment. Within the 465 organic–contaminant analytical space, 49 (27 % of detected) exceeded the EAR = 0.001 screening–level for potential molecular–level effects (Corsi et al., 2019) at least once across all studies. Approximately 72 % (184) of samples had $\sum_{\text{EAR}}$ greater than the 0.001 screening-level, with 47 %, 95 %, and 57 % of private-TW, public-TW, and BW samples, respectively, exceeding this level. About 11 % (29) of samples had $\sum_{\text{EAR}}>1$ indicating an elevated probability of molecular-level effects, with 1 %, 20 %, and 10 % of private–TW, public–TW, and BW samples, respectively, exceeding this level.

Multiple pesticides and VOC were primary drivers of $\sum_{\text{EAR}}$ in private–TW samples. Notably, butanol, although detected only in four POU–DW samples (1 Private-TW, 3 BW) collected to date, had EAR ranging 2.6 – 20.6 indicating high probability of molecular effects when present. DBP were common drivers of molecular activity ($\sum_{\text{EAR}}$) in public–TW and BW samples, with substantial additional contributions by diverse organics, comprising primarily pesticides and other VOC, including historical DW–contaminant concerns like the industrial solvent trichlorethylene and the petroleum hydrocarbon *o*-xylene.

The second EAR screening approach (EAR_MED_) employed HTTK modeling to estimate the *in vivo* molecular-level exposure or measured equivalent dose (MED). Among the 184 organics detected across all studies, 81 (44 %) had reliable ACC data in ToxCast, HTTK–modeling information, and at least one exposure resulting in an individual EAR_MED_ ≥ 0.00001 and were included in the $\sum_{\text{EAR-MED}}$ assessment. Within the 465 organic–contaminant analytical space, 22 ( 12 % of detected) exceeded the EAR_MED_ = 0.001 screening–level for potential molecular–level effects (Corsi et al., 2019) at least once across all studies. Approximately 47 % (120) of samples had $\sum_{\text{EAR-MED}}$ greater than the 0.001 screening-level, with 35 %, 66 %,and 10 % of private-TW, public-TW, and BW samples, respectively, exceeding this level. About 2 % (6) of samples had $\sum_{\text{EAR-MED}}>1$ indicating an elevated probability of molecular-level effects, with 3 %, 0 %, and 10 % of private–TW, public–TW, and BW samples, respectively, exceeding this level.

Butanol and perfluorohexanoic acid (PFHxA) and to a lesser extent bentazone (herbicide), fluticasone proprionate (corticoid steroid treatment for allergic rhinitis/asthma), and a range of VOC, pesticide and pesticide-degradate, and PFAS compounds were primary drivers of $\sum_{\text{EAR-MED}}$ in private–TW samples. Consistent with EAR results above, butanol (1 Private-TW, 3 BW) had EAR_MED_ ranging 1.3 – 10.2 again indicating high probability of molecular effects when present. PFHxA, the DBP 1,1,1-trichloropropanone, and the herbicide 2,4-dichlorphenoxyacetic acid (2,4–D) were common drivers of $\sum_{\text{EAR-MED}}$ in public–TW, with additional contributions by a range of VOC, pesticide and pesticide–degradate, and PFAS compounds. Butanol (3 samples) and isopropanol (2 samples) were primary drivers of $\sum_{\text{EAR-MED}}$ in BW samples.

Consistent with the apical health–benchmark $\sum_{\text{TQ}}$ screening above, the $\sum_{\text{EAR}}$ and $\sum_{\text{EAR-MED}}$ results indicate that simultaneous exposures to multiple organic contaminants of potential concern are common across all DW supplies, likewise emphasizing the need for improved understanding of the adverse human-health implications of long-term exposures to low–level organic–contaminant mixtures across all three distribution pipelines (public–TW, private-TW, BW). These molecular-effects risk results corroborate the need for improved source-water protection, monitoring and characterization, and treatment options across all three pipelines.

## 4. Study limitations

Several limitations merit consideration when interpreting the results of this meta–analysis and of the underlying individual studies. First, studies by this group are conducted modularly to ensure community–scale relevance and priorities while maintaining a broad-scope core analytical toolbox that ultimately supports a national perspective in aggregate. The total number of samples analyzed to date across all studies is limited (98 private-well TW, 126 public–supply TW, and 30 BW) due to high per–sample costs associated with the extensive target analytical approach; further latitudinal (geospatial variability) and longitudinal (temporal variability) assessment is required to fully inform the range of POU-DW exposures in the US and globally, across all three supplies. Second, the target analytical scope, while extensive and environmentally informative, is only a fractional indicator of the estimated 350,000 anthropogenic chemicals in commercial production (Wang et al., 2020) (not including environmental transformation products and degradates) and, thus, potentially present in ambient DW source waters; accordingly, the exposure and associated risk results reasonably may be interpreted as potential orders-of-magnitude underestimates. Third, the target analytical scope does not include some important emerging POU-DW contaminant concerns. For example, micro/nanoplastics–contaminants in POU-DW are rapidly growing concerns due to documented ingestion, biological uptake, translocation (including fetal), and toxicity (Cox et al., 2019; Kannan and Vimalkumar 2021; Medley et al., 2023); orders-of-magnitude higher micro-/nano-plastics contaminant levels have been reported in BW compared to TW (Cox et al., 2019; Kannan and Vimalkumar 2021; Mason et al., 2018; Mitrano et al., 2021; Qian et al., 2024). Fourth, as discussed the $\sum_{\text{TQ}},\sum_{\text{EAR}}$ and $\sum_{\text{EAR-MED}}$ approaches employed herein are limited by available weighting–factors (human–health benchmarks, ToxCast ACC, and ToxCast ACC and *httk* information, respectively) and estimate mixture effects assuming approximate concentration addition (e.g., Cedergreen et al., 2008; Ermler et al., 2011; Stalter et al., 2020). Fifth, cumulative risks ($\sum_{\text{TQ}}$) were compared across supply chains assuming equivalent lifetime consumption (i.e., potential differences in individual and daily consumption were not considered). Sixth, to provide a precautionary lower-bound estimate of *in vivo* adverse–effect levels, EAR and EAR_MED_ were aggregated across all ToxCast endpoints without restriction to recognized modes of action (Paul Friedman et al., 2020), but this approach, while reasonable for screening and prioritization, may not precisely reflect apical effects and should not be used to infer health risk (Blackwell et al., 2017; Schroeder et al., 2016). Seventh, the PFAS results included herein reflect method reporting limits circa and in one case (PFOS) exceeding the EPA PFAS Rule MCL, generally are not sufficient to inform risks to presumptive most sensitive populations (e.g., MCLG of “zero” for PFOS and PFOA), and, consequently, should be considered underestimates of POU-DW PFAS detections and risks; improved methods with reporting limits ≤ 1 ng L^−1^ are available now (e.g., (U.S. Environmental Protection Agency 2024a)). Lastly, MCLG values of “zero” were set to 0.1 μg L^−1^ for metals, DBP, and other VOC and to 0.0001 μg L^−1^ for PFOS and PFOA to avoid overinflating TQ calculations; however, this substitution may not be sufficiently protective for some molecularly-triggered, self–propagating toxicities, including potential carcinogenicity and endocrine– or immune–system responses.

## 5. Conclusions/Implications

Drinking water, in the US is delivered to consumers via private–TW, public–TW, and BW, each with specific logistical, infrastructure, regulatory, and commercial profiles, but, as shown herein, all are confronted by anthropogenic water–quality concerns. Water is a biological necessity and, consequently, a particularly vulnerable route of human exposure to environmental chemical/biological hazards (human-health risk vector). Accordingly, drinking water is a critical nexus of anthropogenic–contaminant cause and human effects and a high leverage point for individual and community–level engagement in risk mitigation at various points along the anthropogenic–contaminant cause-effect continuum, including, for example: chemical design, production, use, and disposal; source-water protection; drinking-water supply selection; and community and point–of-use treatment.

Source-water and infrastructure concerns are intrinsic to all three DW supply chains. However, differences in US regulation, monitoring, community–right–to–know practices, and, notably, commercial marketing between private-TW, public-TW, and BW are fundamental contributors to and, in the latter case, primary drivers of public perceptions of DW-supply quality and safety and, consequently, directly impact consumer decision–making and water-quality and sustainability engagement (Bouhlel et al., 2023; Cohen and Ray 2018; Debbeler et al., 2018; Doria et al., 2009; Hawkins 2017; Jaffee and Newman 2013; Jaffee 2020; Meehan et al., 2020a; Meehan et al., 2020b; Opel 1999; Saylor et al., 2011; U.S. Government Accountability Office 2009; Wilk 2006). Because perceptions of relative safety and acceptable risk (risk tolerance) vary widely individually and across communities and cultures (Hrudey et al., 2006; Hrudey 2009), intercomparable contaminant exposure and human-health risk assessment datasets, which realistically reflect real-world environmental–contaminant complexity (Bradley et al., 2017; Glassmeyer et al., 2017; Moschet et al., 2014; Rosenblum et al., 2024) and quantitatively address all three DW supplies (private-TW, public-TW, BW), are essential to 1) eliminate perception-based, public–engagement barriers raised by extant disparities in data–availability and commercial messaging, 2) refocus attention on the fundamental anthropogenic–contaminant challenge to sustainable DW supply, and 3) provide an objective scientific foundation for corresponding decision-making at multiple levels including regulatory agencies, water suppliers, communities, and, ultimately, end-users.

This meta-analysis demonstrates that elevated human–health risk from simultaneous exposures to multiple drinking-water contaminants is a common challenge across all DW supplies, emphasizing the need for improved understanding of the adverse human-health implications of long-term exposures to low–level inorganic-/organic-contaminant mixtures across all three distribution pipelines. The results demonstrate that considerable contaminant–exposure variability exists within private–TW, public–TW, and BW supplies, with generally comparable ranges in cumulative contaminant concentrations observed to date across all three distribution pipelines. Thus, the results illustrate the importance of continued systematic, quantitative assessments of realistically-broad contaminant exposures and associated bioactivities in POU-DW from all three distribution pipelines to 1) inform public-health research into the role of DW–contaminant–mixture exposures, including low-level exposures, in adverse human-health outcomes, 2) identify and develop DW–contaminant mitigation strategies, and 3) support models of DW–contaminant exposures and related risks at the point of consumption.

The results to date do not support market-driven perceptions of BW as systematically higher quality than public-TW, belying the risk–avoidance messaging underlying the rapid growth in BW consumption (Bouhlel et al., 2023; Cohen and Ray 2018; Hawkins 2017; Jaffee and Newman 2013; Jaffee 2020; Opel 1999; Wilk 2006) and emphasizing the universal need for improved source–water protection, monitoring and characterization, and treatment options across all three pipelines. Ironically because individually-packaged BW in the US is primarily sourced (e.g., >63 % market share in 2014 (Food and Water Watch.org 2018); >64 % of US BW facilities in 2022 (Buchwald et al., 2023; Corson-Dosch et al., 2023)) from public–TW, the theoretical water–quality benefit of BW consumption stems from additional treatment to further reduce concentrations of contaminants (including those contaminants introduced by public–supply disinfection; i.e., DBP). However, the same benefit may be achieved by investment in improved treatment and processes at the DW treatment facility or in properly–maintained POE or POU treatment in the home, workplace, or public facility (e.g., airport) at lower per annum cost and without the plastic-waste (Arp et al., 2021; Bouhlel et al., 2023; Hawkins 2011; Persson et al., 2022; Villarrubia-Gómez et al., 2018) and plastic–attributable US disease burden (Trasande et al., 2024) concerns intrinsic to individually–packaged–BW consumption. POU treatment offers the additional advantage of removing pathogen-suppressing, residual disinfectant immediately before consumption, simultaneously addressing DW–DBP–exposure and DW-pathogen regrowth and exposure concerns.

## Supplementary Material

Supplement1

## Figures and Tables

**Table 1:** Comparison of regulatory, financial, infrastructure, and waste-stream characteristics of private–tapwater, public–tapwater, and bottled–water drinking-water distribution pipelines in the United States.^a.^

Metric		Private Tapwater	Public Tapwater	Bottled Water
**Regulatory**	**Authority**	None	SDWA NPDWR	FDCA SOQ (as food)
	**Agency**	None	EPA (state primacy)	FDA (state primacy)
	**Compliance**	Homeowner (if any)	EPA (state primacy)	FDA (state primacy)
	**Monitoring**	Homeowner (if any)	DWTP (routine, frequent)	BWP (site visit/for cause)
	**Treatment**	Homeowner (if any)	Yes	Varies
	**Reporting**	None	Annual Consumer Report, SDWA Violations page	Bottle label (additional information on request)
**Consumer Cost**	**Cost Type**	None	Utility Fee	Per package cost
	**Volumetric Cost**	None	Low	High
**Organizational**	**Profit**	No	No (public nonprofit)^b^	Yes
			Yes (private for-profit)	
	**Marketing**	No	No	Extensive
**Infrastructure**	**Source water**	Groundwater	Surface water/Groundwater	Public tapwater^c^/Groundwater
	**Type**	Private well, Premise plumbing	Distribution system, Premise plumbing	Commercial packaging
	**Maintenance**	Homeowner	Covered by Utility Fee	Per package cost
	**Monitoring**	Homeowner (rare)	Covered by Utility Fee	Per package cost
**Waste Stream**	**Water**	Yes	Yes	Yes (tapwater)^d^
	**Package**	No	No	Yes (plastic)

a [BWP – bottled–water plant; DWTP – drinking–water treatment plant; EPA – United States Environmental Protection Agency; FDA – United States Food and Drug Administration; FDCA – Food, Drug, and Cosmetics Act; NPDWR – National Primary Drinking Water Regulations; SDWA – Safe Drinking Water Act].

b [some privately-owned water utilities are operated as nonprofit].

c [more than half of bottled water consumed in US is sourced from public supply, which in turn is sourced from surface water and groundwater].

d [bottled water consumption for drinking/cooking generally represents a negligible reduction in total residential or workplace tapwater use; comparable water-waste profiles expected regardless of drinking-water pipeline].

## Data Availability

Data will be made available on request.
